# EpCAM deficiency causes premature aging of intestinal epithelium via hyperactivating mTORC1 pathway

**DOI:** 10.1002/ctm2.903

**Published:** 2022-06-09

**Authors:** Zili Lei, Lei Chen, Yanyan Liu, Yanhong Yang, Guibin Chen, Wanwan Liu, Ya Nie, Yuting Lei, Fengxue Tong, Li Huang, Huijuan Wu, Lanxiang Yang, Xueying Zhang, Changyuan Yang, Jiamin Zhu, Jiao Guo

**Affiliations:** ^1^ Guangdong Metabolic Diseases Research Center of Integrated Chinese and Western Medicine Guangdong Pharmaceutical University Guangzhou People's Republic of China; ^2^ The First Affiliated Hospital (School of Clinical Medicine) Guangdong Pharmaceutical University Yue‐Xiu District Guangzhou People's Republic of China; ^3^ School of Traditional Chinese Medicine Guangdong Pharmaceutical University, Guangzhou Higher Education Mega Center Guangzhou People's Republic of China

Dear Editor,

1

We reported a new role of epithelial cell adhesion molecule (EpCAM) on maintaining the longevity of intestinal epithelial cells (IECs) via regulating the TSC1/mTORC1/Sirtuins pathway. EpCAM has been used as a cell surface marker of various cancer tissues and many kinds of stem cells,^1^, ^2^, ^3^ but its functions in cancers and stem cells were less studied. The deficiency of EpCAM causes the breakdown of the homeostasis of intestinal epithelium, which is called the congenital tufting enteropathy (CTE) in patients.^4^ However, the function of EpCAM in IECs was still unclear.

Intestines from E18.5 embryos and postnatal pups of wild type (WT) and EpCAM^–/–^ mice were first compared. Similar to previously reported,^1^ the EpCAM^–/–^ pups showed no bodyweight gain, serious diarrhoea and damaged intestines (Figures 1A–C, S1A–C). Genes for intestinal abundant keratins, including *Krt8*, *Krt18*, *Krt19*, *Krt20* and *Krt23*, were remarkably upregulated in the small intestines of mutant embryos and pups (Figures 1D,E, S1F). These results confirmed that the homeostasis of intestinal epithelium was seriously affected in EpCAM^–/–^ mice.

**FIGURE 1:**
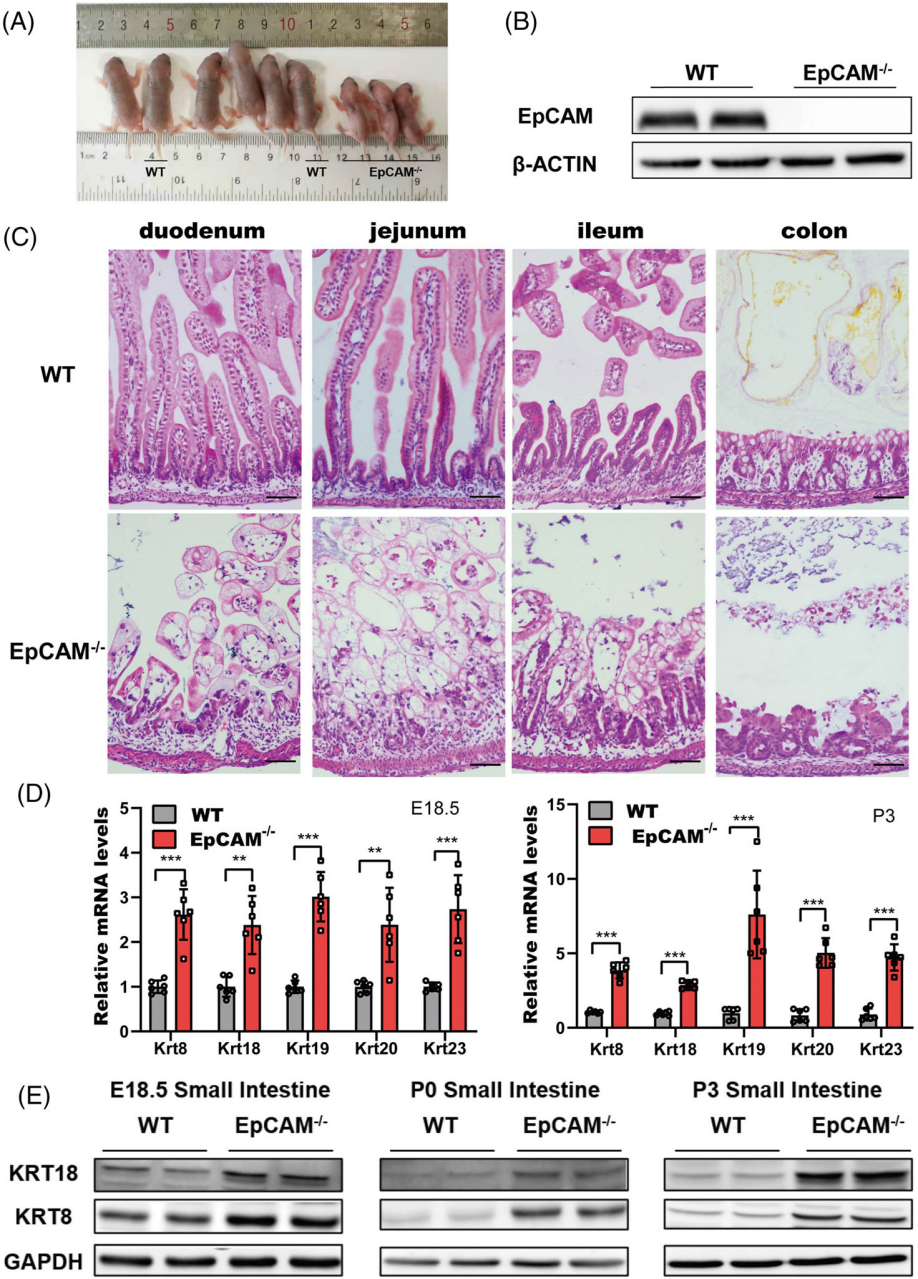
The intestinal epithelium of EpCAM^–/–^ mice was severely damaged and keratinized. (A) Nine pups of littermate from EpCAM^+/–^ parents at the P3 stage. The genotype of 2 WT and 3 EpCAM^–/–^ pups were indicated, and the other 4 pups were heterozygotes. (B) Representative western blot images showed the EpCAM protein level in the small intestines of WT and EpCAM^–/–^ mice at P3 stage. (C) Representative hematoxylin and eosin (H&E) staining images of duodenum, jejunum, ileum and colon tissues from WT and EpCAM^–/–^ mice at P4 stage. Scale bar, 50 μm. (D) Graphs showed the relative mRNA expression levels of the indicated genes in small intestines from WT and EpCAM^–/–^ mice at E18.5 and P3 stages, respectively (WT, *n* = 6; EpCAM^–/–^, *n* = 6). ***P < 0.001, **P < 0.01 compared with WT group. (E) Representative western blot images of KRT8 and KRT18 proteins in small intestines from WT and EpCAM^–/–^ mice at E18.5, P0 and P3 stages, respectively (6 mice in each group for 3 times independent experiments)

To investigate the function of EpCAM in IECs, the expression of telomerase reverse transcriptase (TERT) and telomerase RNA component (TERC), components of Telomerase,^5^ was tested. TERT was noticeably downregulated in the intestines of EpCAM^–/–^ mice compared to the WT controls (Figures 2A–C, S2A–C). The transcription of *Terc* was only considerably reduced in the small intestines of mutant P0 pups (Figures 2A, S2A). Telomeres are bound by Shelterin which includes six subunits: TRF1, TRF2, POT1, TPP1, RAP1 and TIN2.^5^ TRF1 remarkably decreased in small intestines of mutant mice (Figures 2D, S2D). 53BP1, which can be recruited to the damaged telomeres,^6^ was upregulated in the small intestines from mutant mice at the posttranscriptional level (Figures 2D, S2D). EpCAM^–/–^ Caco‐2 cells were generated for in vitro exploration (Figure S1D,E). TERT and TRF1 were significantly reduced in EpCAM^–/–^ Caco‐2 cells (Figure S2G‐J). However, the protein levels of TRF2 and 53BP1 were still normal in EpCAM^–/–^ Caco‐2 cells (Figure S2I‐J). These results indicated that EpCAM mutation would affect the length of telomeres in IECs and induce premature aging of intestinal epithelium.

**FIGURE 2:**
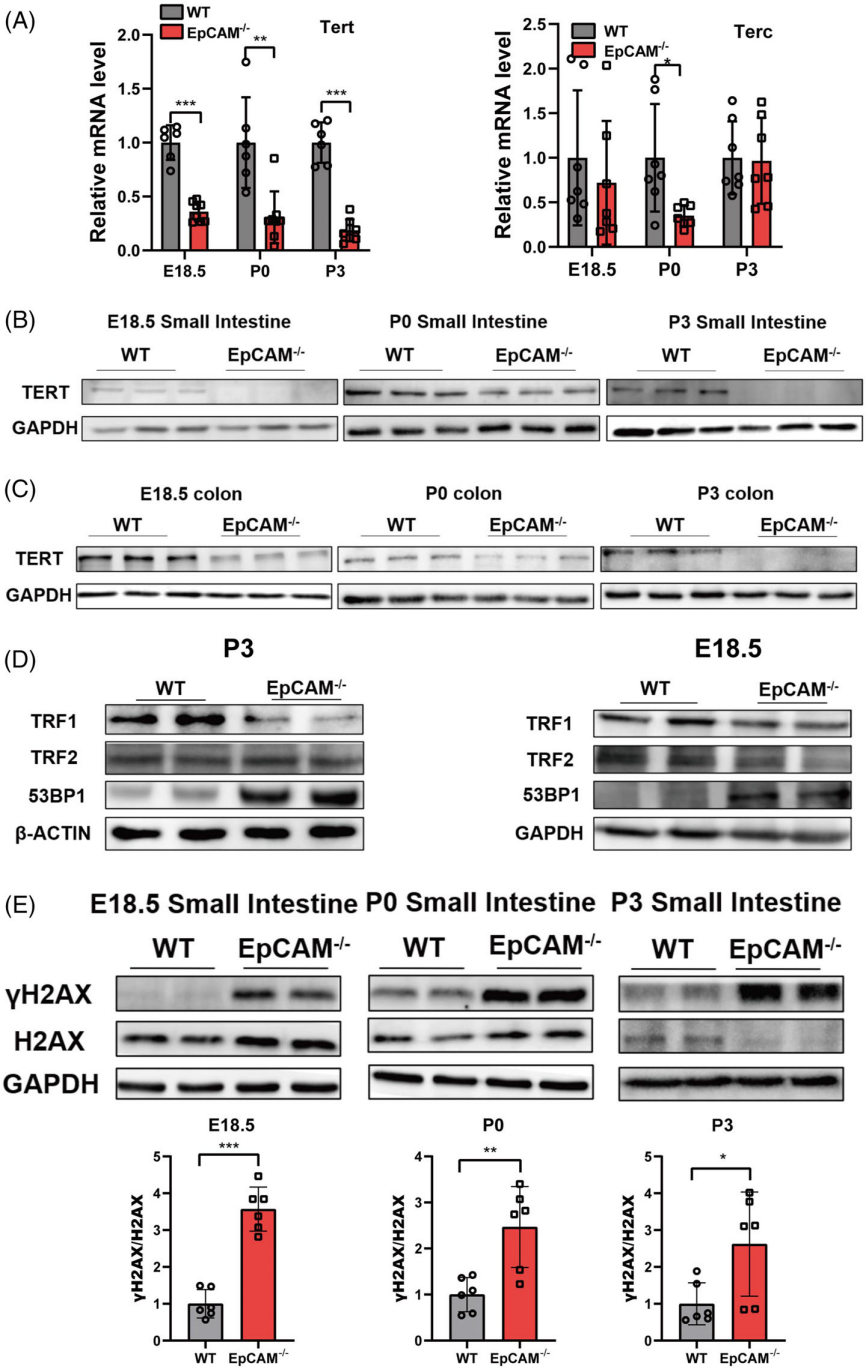
EpCAM deficiency affected telomeres and caused accumulation of unrepaired DNA double‐strand breaks in the intestines of mice. (A) The mRNA expression levels of *Tert* and *Terc* in the small intestines of WT and EpCAM^–/–^ mice at E18.5, P0 and P3 stages, respectively (Tert: WT *n* = 6, EpCAM^–/–^, *n* = 8; Terc: WT *n* = 7, EpCAM^–/–^ *n* = 7). ***P < 0.001, **P < 0.01, *P < 0.05 compared with WT group. (B) Representative western blot images of TERT in small intestines from WT and EpCAM^–/–^ mice at E18.5, P0 and P3 stages, respectively (6 mice in each group for two times independent experiments). (C) Representative western blot images of TERT in colon from WT and EpCAM^–/–^ mice at E18.5, P0 and P3 stages, respectively (6 mice in each group for two times independent experiments). (D) Representative western blot images of TRF1, TRF2 and 53BP1 in the small intestines of WT and EpCAM^–/–^ mice from E18.5 and P3 stages, respectively (6 mice in each group for three times independent experiments). (E) Western blot analysis of the levels of γH2AX and H2AX in the small intestines from WT and EpCAM^–/–^ mice at E18.5, P0 and P3 stages, respectively. Lower panels: quantification data. Six mice in each group for three times independent experiments. ***P < 0.001, **P < 0.01, *P < 0.05 compared with WT group

The accumulation of unrepaired DNA double‐strand breaks (DSBs), marked by the increase of γH2AX,^7^ was assessed. At E18.5 and P0 stages, the ratio of γH2AX to H2AX was considerably increased in the small intestines of mutant mice (Figure 2E). For P3 mice, the expression level of H2AX became reduced in the small intestines of mutant pups, but γH2AX still significantly increased in them (Figure 2E). γH2AX also significantly increased in EpCAM^–/–^ Caco‐2 cells (Figure S3A). These results demonstrated that EpCAM mutation increased DNA damage in IECs both in vivo and in vitro. The accumulation of DNA damage is one aging hallmark.^8^

To explore the mechanisms of EpCAM deficiency on accelerating the aging of IECs, members of Sirtuin family^8^ were checked and they all downregulated in the small intestines from mutant mice at transcriptional or posttranscriptional levels, especially SIRT3, SIRT6 and SIRT7 (Figures 3A,B, S4A‐B, S4E, S6A). SIRT1, SIRT5, SIRT6 and SIRT7 were also significantly downregulated in the EpCAM^–/–^ Caco‐2 cells (Figure S4C,D,F). After administration of MDL‐800, activator of SIRT6,^9^ to EpCAM^–/–^ pups, the expression of Sirtuins significantly increased in the small intestines of them (Figures 3C,D, S6B). MDL‐800 could significantly upregulate the expression of *Tert* in the intestines of mutant pups (Figures 3E,F, S6C). γH2AX was remarkably decreased in the intestines of mutant pups administrated with MDL‐800 (Figures 3G, S6D). These results demonstrated that the downregulation of the expression of Sirtuins might be one of the mechanisms of the premature aging of IECs from EpCAM^–/–^ mice.

**FIGURE 3:**
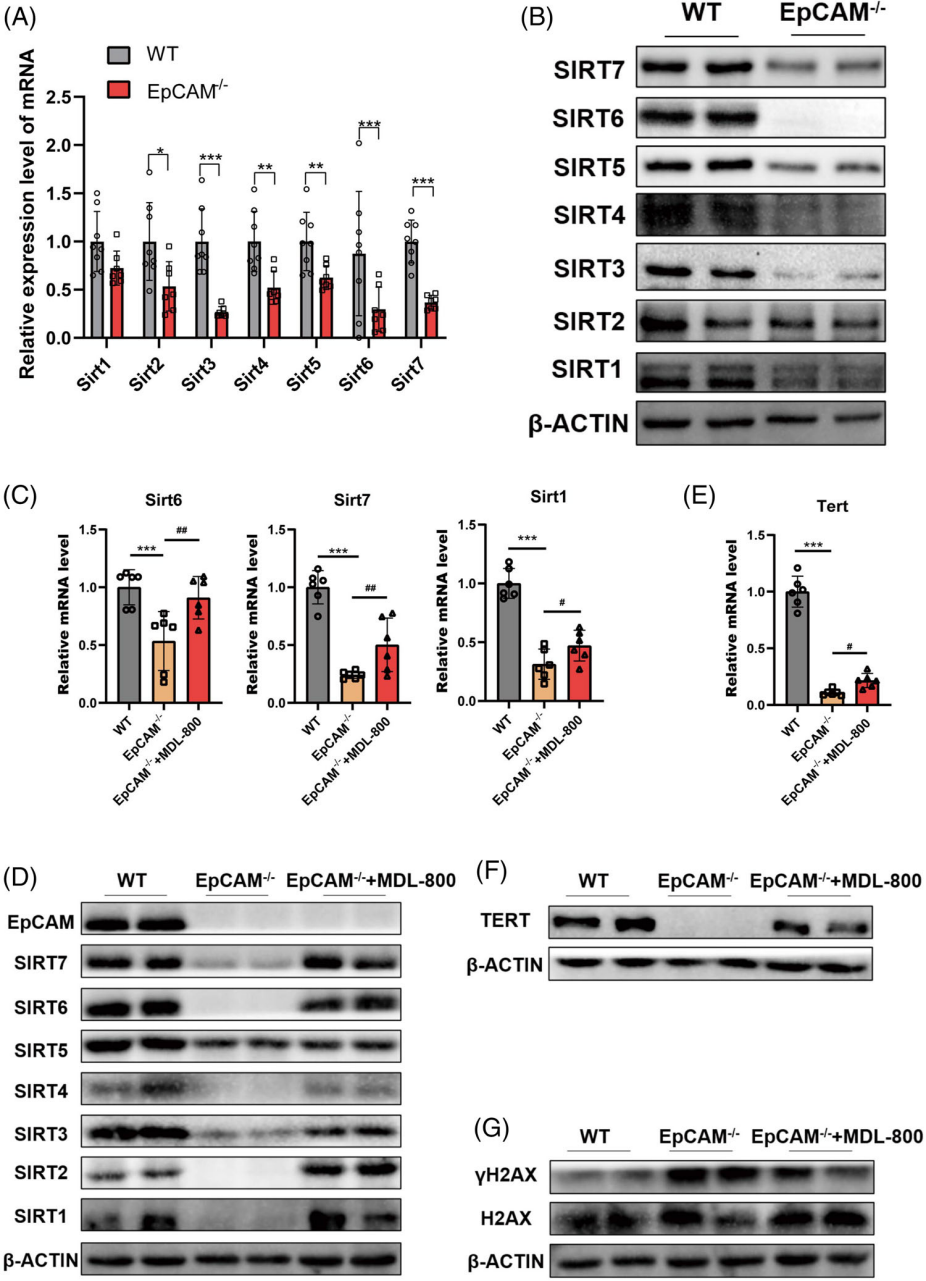
Downregulation of sirtuins caused premature aging of the intestinal epithelial cells of EpCAM^–/–^ Mice. (A) The mRNA expression levels of Sirt1‐7 in the small Intestines of WT and EpCAM^–/–^ pups at P3 stage (Sirt1‐7: WT, *n* = 8; EpCAM^–/–^, *n* = 7). ***P < 0.001, **P < 0.01, *P < 0.05 compared with WT group. (B) Representative western blot images of SIRT1‐7 in the small intestines of WT and EpCAM^–/–^ pups at P3 stage (6 mice in each group for three times independent experiments). (C) Graphs showed the relative mRNA expression levels of Sirt6, Sirt7 and Sirt1 in the small intestines of WT, EpCAM^–/–^ and MDL‐800 administrated EpCAM^–/–^ mice at P3 stage (*n* = 6 per group). ***P < 0.001 compared with WT group; ^##^ *P* < 0.01, ^#^ *P* < 0.05 compared with EpCAM^–/–^ group. (D) Western Blot results showed the levels of EpCAM and SIRT1‐7 in the small intestines from WT, EpCAM^–/–^ and MDL‐800 administrated EpCAM^–/–^ mice at P3 stage (4 mice in each group for two times independent experiments). (E) Quantitative real time PCR (qPCR) results of Tert from the small intestines of WT, EpCAM^–/–^ and MDL‐800 administrated EpCAM^–/–^ mice at P3 stage (*n* = 6 per group). ***P < 0.001 compared with WT group; ^#^ *P* < 0.05 compared with EpCAM^–/–^ group. (F) Representative western blot image of TERT in the small intestines from WT, EpCAM^–/–^ and MDL‐800 administrated EpCAM^–/–^ mice at P3 stage (4 mice in each group for two times independent experiments). (G) Representative western blot images of γH2AX and H2AX in the small intestines from WT, EpCAM^–/–^ and MDL‐800 administrated EpCAM^–/–^ mice at P3 stage (4 mice in each group for two times independent experiments)

To study signal pathways related to the premature aging of IECs caused by EpCAM deficiency, the mTORC1 pathway^10^ was checked. TSC1, TSC2 and p‐TSC2, but not p‐TSC2/TSC2, were all considerably decreased in the small intestines of mutant pups (Figures 4A–C, S7A). The increase of p‐mTOR, p‐mTOR/mTOR, p‐S6, p‐S6/S6 and p‐4EBP1 confirmed the hyperactivation of the mTORC1 pathway in the intestines of EpCAM^–/–^ pups (Figures 4C, S7A). TSC1 was also significantly decreased in EpCAM^–/–^ Caco‐2 cells, and the ratios for p‐mTOR/mTOR and p‐S6/S6 remarkably increased in EpCAM^–/–^ cells (Figures S5A,B, S8A). These results illustrated that the mTORC1 pathway was hyperactivated in EpCAM^–/–^ IECs. Rapamycin was administrated to EpCAM^–/–^ pups to reduce the hyperactivation of mTORC1 in IECs of them (Figures 4D,E, S7B). The small intestinal epithelium of P3 EpCAM^–/–^ pups was slightly improved after administration of rapamycin (Figure 4F). The protein level of TERT increased and the level of γH2AX reduced in the intestines of rapamycin administrated P3 mutant mice (Figure 4G, S7C). The protein levels of SIRT1, SIRT4, SIRT5, SIRT6 and SIRT7 were also rescued in the intestines of P3 mutant mice by rapamycin (Figure 4G, S7C). Rapamycin treatment also significantly resumed the expression of *Tert* and Sirtuins and reduced the level of γH2AX in EpCAM^–/–^ Caco‐2 cells (Figures S5C–F, S8B–D). These results demonstrated that EpCAM deficiency caused the premature aging of IECs via hyperactivating the mTORC1 pathway both in vivo and in vitro. However, administration of MDL‐800 could not inhibit the hyperactivation of mTORC1 pathway in the intestines of EpCAM^–/–^ pups (Figures 4H, S7D), indicating that mTORC1 might be upstream of SIRTs in IECs.

**FIGURE 4:**
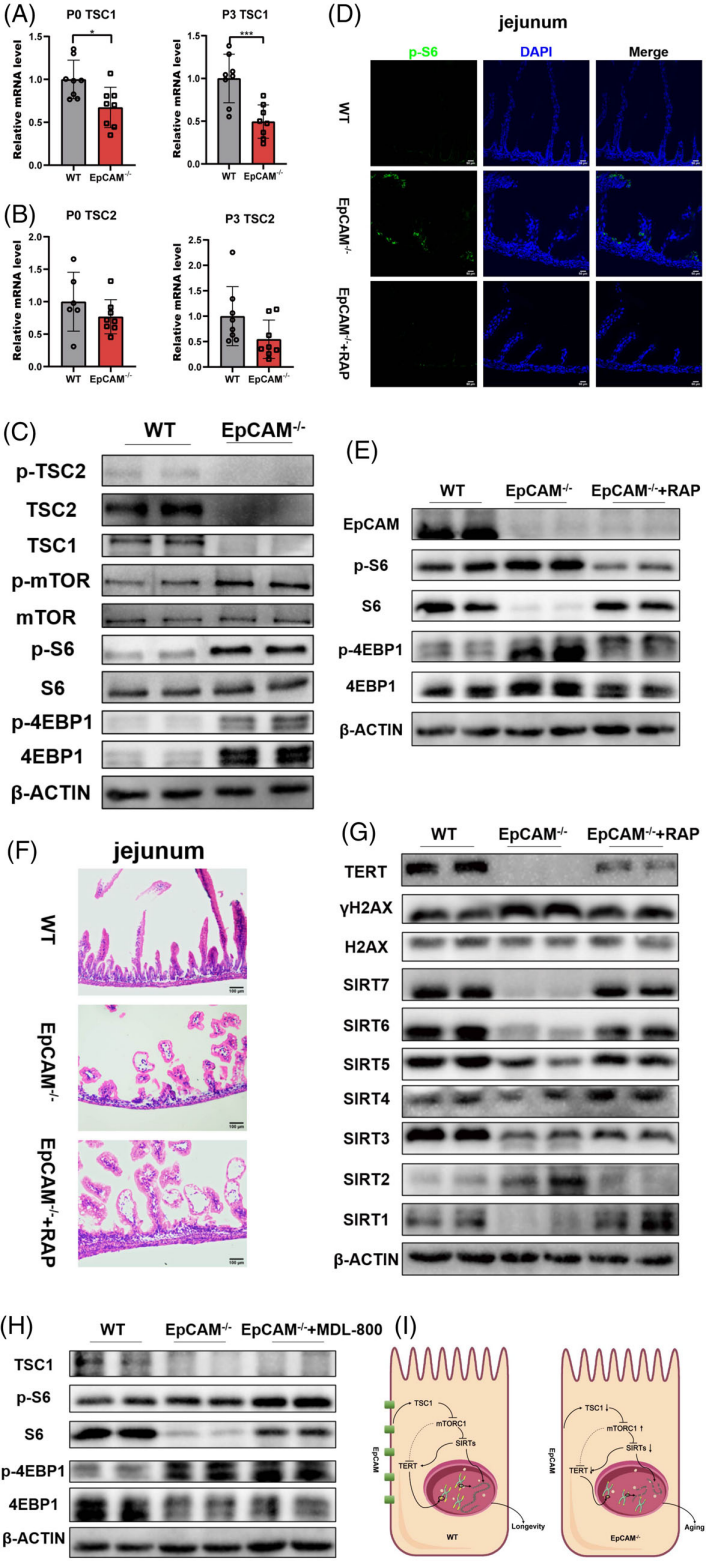
Hyperactivation of mTORC1 induced the premature aging of the intestinal epithelial cells of EpCAM^–/–^ mice. (A) Graphs showed the relative mRNA expression levels of TSC1 in small intestines of WT and EpCAM^–/–^ pups from P0 and P3 stages, respectively (*n* = 8 per group). ***P < 0.001, *P < 0.05 compared with WT group. (B) The mRNA expression levels of TSC2 in the small intestines of WT and EpCAM^–/–^ mice at P0 and P3 stage, respectively (P0: WT, *n* = 6; EpCAM^–/–^, *n* = 8. P3: WT, *n* = 8; EpCAM^–/–^, *n* = 8). **P < 0.01 compared with WT group. (C) Western blot results showed the levels of p‐TSC2, TSC2, TSC1, p‐mTOR, mTOR, p‐S6, S6, p‐4EBP1 and 4EBP1 in the small Intestines of WT and EpCAM^–/–^ pups at P3 stage (6 mice in each group for three times independent experiments). *P < 0.05, **P < 0.01. (D) Images of the immunofluorescence staining of p‐S6 in sections of jejunum from WT, EpCAM^–/–^ and RAP administrated EpCAM^–/–^ pups at P3 stage. Scale bar, 50 μm. (E) Western blot results showed the levels of EpCAM, p‐S6, S6, p‐4EBP1 and 4EBP1 in the small intestines from WT, EpCAM^–/–^ and RAP administrated EpCAM^–/–^ pups at P3 stage (4 mice in each group for two times independent experiments). (F) Images of H&E staining of jejunum from WT, EpCAM^–/–^ an RAP administrated EpCAM^–/–^ pups at P3 stage. (G) Western blot results showed the levels of TERT, γH2AX, H2AX and SIRT1‐7 in the small intestines from WT, EpCAM^–/–^ and RAP administrated EpCAM^–/–^ pups at P3 stage (4 mice in each group for two times independent experiments). (H) Western blot results showed the levels of TSC1, p‐S6, S6, p‐4EBP1 and 4EBP1 in the small intestines from WT, EpCAM^–/–^ and MDL‐800 administrated EpCAM^–/–^ mice at P3 stage (4 mice in each group for two times independent experiments). (I) Schematic model of the signalling pathway that EpCAM maintains the longevity of intestinal epithelial cells

In summary, EpCAM deficiency causes the premature aging of IECs via TSC1/mTORC1/sirtuins pathway (Figure 4I). Therefore, mTORC1 and SIRT6 are potential targets for treating the premature aging of IECs of CTE patients. The highly expressed EpCAM might contribute to maintain the genomic stability and the length of telomeres in various stem cells and cancers.

## CONFLICT OF INTEREST

The authors declare that there is no conflict of interest that could be perceived as prejudicing the impartiality of the research reported.

## Supporting information




**Figure S1** EpCAM was successfully knockout in the intestinal epithelial cells both in vivo and in vitro
**Figure S2** EpCAM deficiency affected the compositions of telomerase and telomeres in the intestinal epithelial cells both in vivo and in vitro
**Figure S3** EpCAM deficiency caused accumulation of unrepaired DNA double‐strand breaks in caco‐2 cells
**Figure S4** EpCAM deficiency affected the expression of members of sirtuin family in the intestines of E18.5 embryonic mice and caco‐2 cells
**Figure S5**. Hyperactivation of mTORC1 induced the premature aging of EpCAM^‐/‐^ Caco‐2 cells
**Figure S6** Quantitative analysis of the western blot results in Figure 3
**Figure S7** Quantitative analysis of the western blot results in Figure 4
**Figure S8** Quantitative analysis of the western blot results in Figure S5
**Table S1** Sequences of primers used for qPCR
**Table S2** The primary and secondary antibodies used for western blot
