# Supplementary material for: EpCAM deficiency causes premature aging of intestinal epithelium via hyperactivating mTORC1 pathway
Source: Clin Transl Med. 2022 Jun 9;12(6):e903. doi: 10.1002/ctm2.903 (PMC9178350; doi:10.1002/ctm2.903)

**Supporting information**

**Materials and methods**

**Mice**

All animal experiments were approved by the Committee on Laboratory Animal Care and Use of Guangdong Pharmaceutical University and conformed to NIH guidelines for animal welfare. EpCAM knockout mice were generated previously using CRISPR/Cas9 technology. ^1^ Mice were housed in the SPF mouse facility at 25oC, 60-65% humidity and 12hr light-dark cycle, with free access to water and food. Heterozygous males and females were mated to get embryos and pups for experiments. E18.5 embryos were collected from pregnant heterozygous females. Intestinal tissues were harvested from E18.5 embryos, P0, P3 and P4 pups respectively.

The oral administration of 5mg/kg rapamycin (VETEC, V900930) or 100mg/kg MDL-800 (Sigma, SML2529) to EpCAM^-/-^ pups was performed at P2 stage, and the second dose was administrated after 24 hours. The considerable volume of 2.5% of DMSO was administrated to control EpCAM^-/-^ pups, WT pups and heterozygotes from the same litter. After 8 hours of the second dose administration, intestinal tissues were harvested for experiments.

**Cell experiments**

Caco-2 cells were purchased from the Cell Resource Center, Shanghai Institutes for Biological Science, Chinese Academy of Sciences. Caco-2 cells were cultured in DMEM medium (Gibco, C11995500BT) containing 10% FBS (Gibco, 10270-106)), 10000U/ml penicillin and 10000μg/ml streptomycin (Gibco, 15140-122), in the 5% CO2 incubator at 37°C.

For knockout EpCAM gene from Caco-2 cells, three sgRNA target sequences (ATTATTCACAAAGCAGTTTA, CAATGCCAGTGTACTTCAGT and GTGCACCAACTGAAGTACAC) for the human EpCAM gene (NM_002354.3) were designed at http://www.rgenome.net/cas-designer/, and a non-silencing sgRNA sequence (ACGGAGGCTAAGCGTCGCAA) was used as negative control (sgEpCAM-NC). sgRNA constructs were synthesized and cloned into Lenti-CAS9-sgRNA-PuroR vector with BsmBI sites (Sangon Biotech). The EpCAM-sgRNA plasmids and NC plasmids were transfected into HEK293T cells using LipoHigh transfection reagent (Sangon Biotech，E607403) according to the manufacturer's instructions. The packaging lentivirus particles expressing EpCAM-sgRNA or NC-sgRNA were harvested after transfection for 72 h, and stored at −80°C for further use. Caco-2 cells were seeded in 3.5cm culture dishes and infected with sgEpCAM lentivirus or sgEpCAM-NC lentivirus at multiplicity of infection (MOI) of 200, supplemented with 5μg/ml polybrene (Sigma-Aldrich, 107689) when the confluency of Caco-2 cells reached 50%. The culture medium was refreshed after 48h, and selected with 0.5μg/ml puromycin (Sangon Biotech, A610593) in culture medium, and then the survived cells were kept in the culture medium containing 0.25μg/ml puromycin. The lentiviral infection efficiency was confirmed via qRT-PCR and Western blot technology.

To inhibit the activation of mTORC1 in EpCAM knockout Caco-2 cells, 200μmol/ml rapamycin (VETEC, V900930) in the culture medium was used to incubate for 48h before the cells were harvested for experiments.

**Histological analysis**

H&E staining was performed as previous report.^2^ Simply, intestinal tissues were fixed with 4% paraformaldehyde in PBS at 4^o^C for overnight, then dehydrated and embedded in paraffin. 4μm sections were stained with hematoxylin for 2 min and eosin for 30 sec. Images were got using the PerkinElmer Automated Quantitative Pathology System.

**Quantitative real-time polymerase chain reaction (qRT-PCR)**

Total RNA from mouse intestines or Caco-2 cells was extracted using Trizol reagent (Invitrogen; Thermo Fisher Scientific, Inc.) which was subjected to reverse transcription through the PrimeScript™ RT Reagent kit (Takara Bio, Inc.) at 37˚C for 15 min and then 85˚C for 5 sec. The sequences of qRT-PCR were listed in Table S1 and the primers were produced by Sangon Biotech Co., Ltd. qPCR was conducted using the SYBR Premix Ex Taq kit (Takara Bio, Inc.) and the LightCycler 480II System (Roche, Inc.). The thermocycling program was 95˚C for 30 sec; and then followed by 40 cycles of 95˚C for 5 sec, 60˚C for 20 sec and 65˚C for 15 sec. Mouse and human GAPDH was used as internal reference genes for mouse intestinal tissues and Caco-2 cells respectively.

**Western blot analysis**

Intestinal tissues or Caco-2 cells were lysed in Radio-Immunoprecipitation Assay lysis buffer containing 1% PMSF and 1% protease inhibitor cocktail (Dalian Meilun Biotechnology co., Ltd.), and then centrifuged at 13,680 x g, 4˚C, 30 min, subsequently the supernatant was harvested. Protein concentration was determined using BCA kit (P0011, Beyotime). Then, equal amounts of protein (30μg) were separated via the SDS-PAGE on the 8-12% gel, and then electrophoretically transferred to the PVDF membrane. The PVDF membrane was blocked with 5% non-fat milk in TBST buffer for 1 hour at room temperature, then incubated with primary antibodies for overnight in 4^o^C, and then incubated with HRP (horseradish peroxidase)-labeled secondary antibodies, and the resultant signals were detected using enhanced chemiluminescence reagent (Bio-Rad Laboratories, Inc.; Cat.No.170-5060). The primary antibodies and secondary antibodies used for western blot were listed in Table S2. The quantification of western blot bands was analyzed via the Lane 1d software (version 5.1.0.0; SageCreation).

**Immunofluorescence staining**

The immunofluorescence staining was performed as previously described.^3^ The intestinal tissues were fixed in 4% paraformaldehyde at 4˚C for overnight, then dehydrated, embedded in OCT compound and sectioned. 7-µm-thick frozen sections were first boiled in 10 mM citric acid (Merck) at pH 6.0 for 5 min, then exposed in goat serum blocking buffer (ZSGB-BIO, ZLI- 9056) to block nonspecific sites for 1h at room temperature, following incubated with primary antibodies in blocking buffer at 4˚C for overnight, and then with secondary antibodies for 1h at room temperature. The primary antibodies included rabbit anti-EpCAM (1:200; Abcam, ab71916) and rabbit anti-p-S6 (1:200; CST, #5364). Immunofluorescence analysis was conducted using Alex Fluor 488-labeled secondary antibodies (Invitrogen). Immunofluorescence images were got by using Olympus confocal microscope.

**Statistical analysis**

Statistical analysis was performed using the SPSS software (version 25.0; IBM Corp.). Unpaired two sample t-test was conducted to determine the difference between groups, and data were expressed as mean ± standard deviation. P-value <0.05 was considered to be significant difference.

**References**

1. Yang Y, Liu S, Lei Z, Chen G, Huang L, Yang F, Lei Y, Liu Y, Yang L, Liu W, Lai L, Guo J (2019). Circular RNA profile in liver tissue of EpCAM knockout mice. INT J MOL MED. 44, 1063-1077.

2. Chen G, Yang Y, Liu W, Huang L, Yang L, Lei Y, Wu H, Lei Z, Guo J (2021). EpCAM is essential for maintenance of the small intestinal epithelium architecture via regulation of the expression and localization of proteins that compose adherens junctions. INT J MOL MED. 47, 621-632.

3. Lei Z, Yang L, Lei Y, Yang Y, Zhang X, Song Q, Chen G, Liu W, Wu H, Guo J (2021). High dose lithium chloride causes colitis through activating F4/80 positive macrophages and inhibiting expression of Pigr and Claudin-15 in the colon of mice. Toxicology. 457, 152799.

**
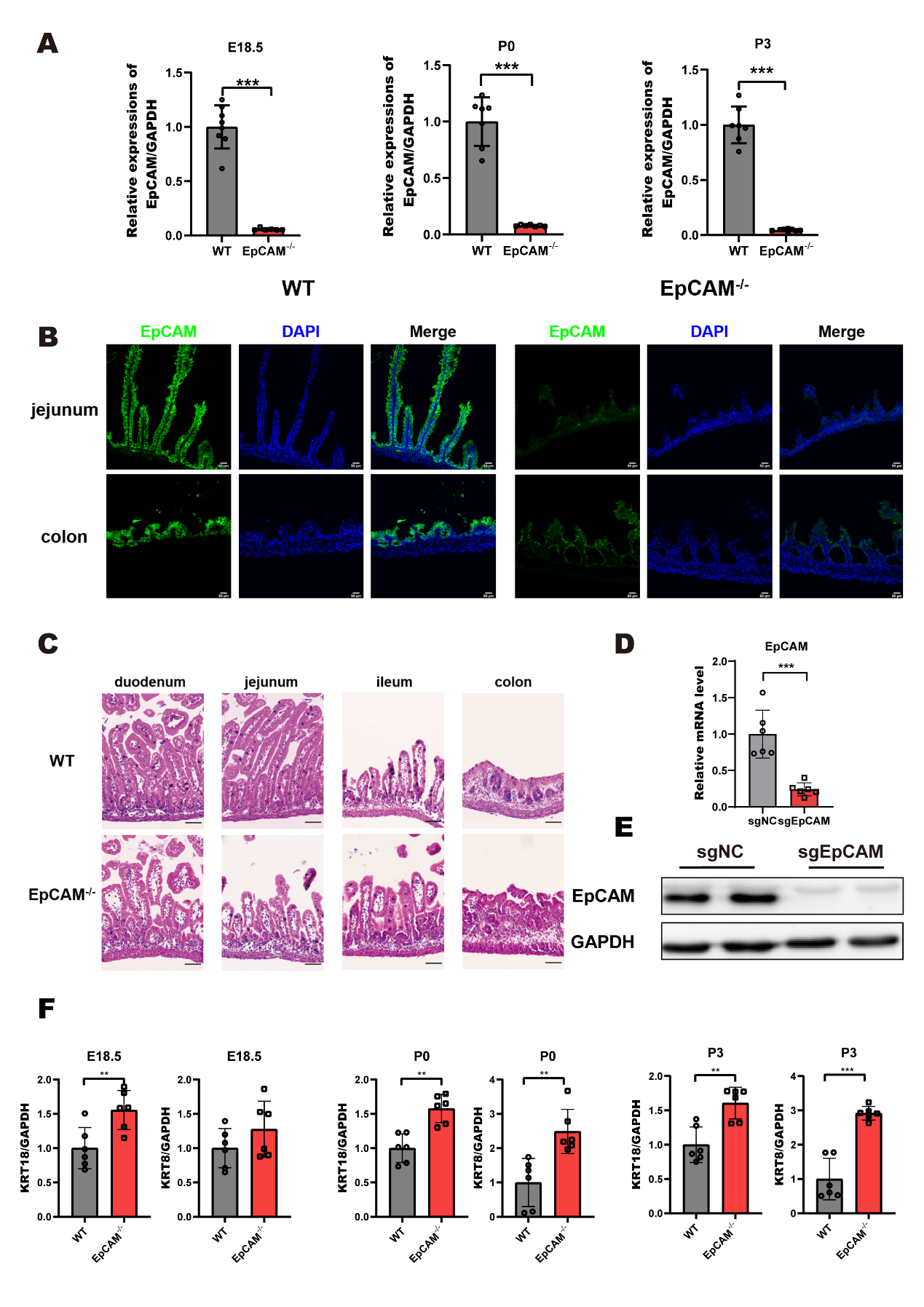
**

**Supplementary Figure Legends**

**Figure S1. EpCAM was Successfully Knockout in the Intestinal Epithelial Cells Both *in vivo* and *in vitro***

**A**. The mRNA expression levels of EpCAM in the small intestines of WT and EpCAM^-/-^ mice from E18.5, P0 and P3 stages respectively (E18.5: WT, n=8; EpCAM^-/-^, n=6. P0: WT, n=7; EpCAM^-/-^, n=7. P3: WT, n=7; EpCAM^-/-^, n=8). ***P<0.001 compared with WT group. **B**. Images of the immunofluorescence staining of EpCAM in sections of jejunum and colon from WT and EpCAM^-/-^ pups at P3 stage. Scale bar, 50μm. **C**. Representative images of hematoxylin and eosin (H&E) staining of duodenum, jejunum, ileum and colon tissues from P0 WT and EpCAM^-/-^ pups. Scale bar, 100μm. **D-E**. The mRNA and protein expression levels of EpCAM in Caco-2 cells from sgNC and sgEpCAM groups were tested through qPCR (n=6) and Western blot. ***P<0.001 compared with sgNC group. **F**. Quantification data of the western blot results of Fig. 1E. 6 mice in each group for 3 times independent experiments. **P<0.01, ***P<0.001 compared with WT group. sgNC, non-specific small guide RNA control; sgEpCAM, EpCAM knockout via CRISPR/Cas9 technology.


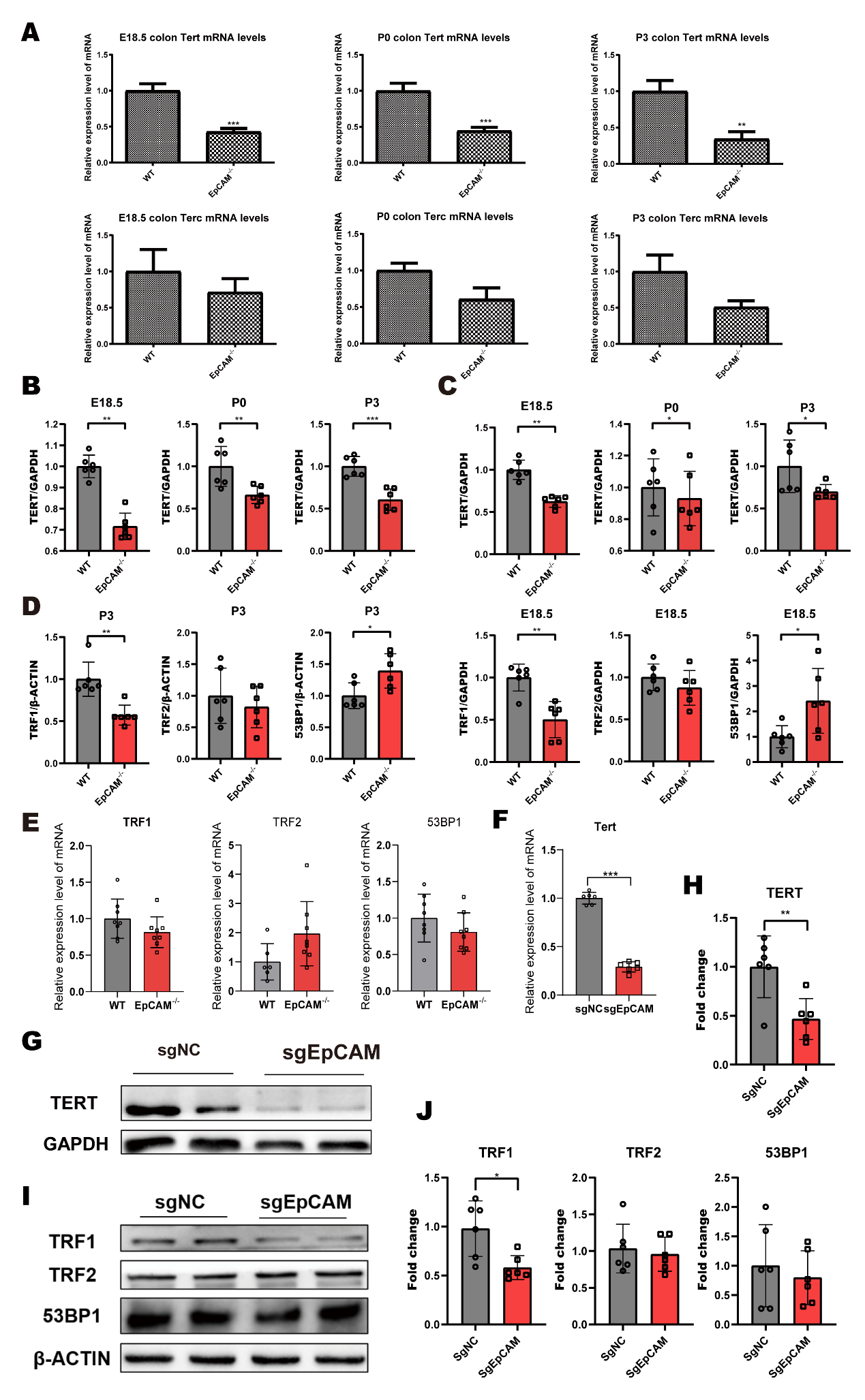


**Figure S2. EpCAM Deficiency Affected the Compositions of Telomerase and Telomeres in the Intestinal Epithelial Cells Both *in vivo* and *in vitro***

**A**. The mRNA expression levels of Tert and Terc in the colon tissues of WT and EpCAM^-/-^ mice at E18.5, P0 and P3 stages, respectively (E18.5: n=8 mice per group; P0 and P3: n=6 mice per group). ***P<0.001, **P<0.01, *P<0.05 compared with WT group. **B-D**. Quantification data of the western blot results of Fig. 2B-D, respectively (B-C: 6 mice in each group for 2 times independent experiments; D: 6 mice in each group for 3 times independent experiments). *P<0.05, **P<0.01, ***P<0.001 compared with WT group. **E**. The mRNA expression levels of TRF1, TRF2 and 53BP1 in the small intestines of WT and EpCAM^-/-^ P3 pups. (TRF1: WT, n=8; EpCAM^-/-^, n=8. TRF2: WT, n=6; EpCAM^-/-^, n=8; 53BP1: WT, n=8, EpCAM^-/-^, n=8). **F**. The mRNA expression levels of Tert in Caco-2 cells from sgNC and sgEpCAM groups were tested through qPCR (n=6). ***P<0.001 compared with sgNC group. **G-H**. Western blot results of TERT in Caco-2 cells from sgNC and sgEpCAM groups (n=6 in each group for 3 times independent experiments). **P<0.01 compared with sgNC group. **I-J**. Western blot results of TRF1, TRF2 and 53BP1 in Caco-2 cells from sgNC and sgEpCAM groups (n=6 in each group for 3 times independent experiments). *P<0.05 compared with sgNC group. sgNC, non-specific small guide RNA control; sgEpCAM, EpCAM knockout via CRISPR/Cas9 technology.


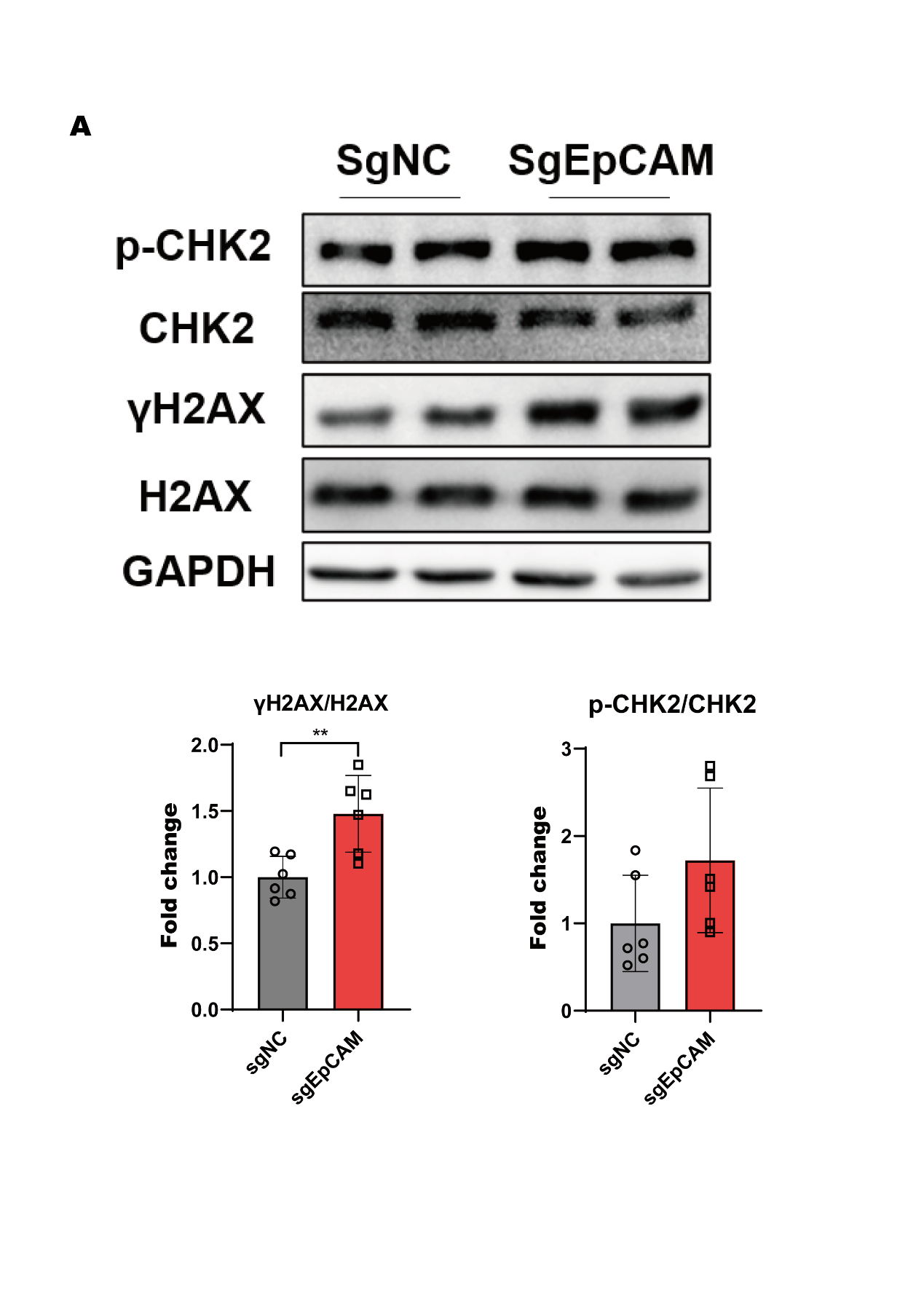


**Figure S3. EpCAM Deficiency Caused Accumulation of Unrepaired DNA Double-Strand Breaks in Caco-2 Cells**

**A**. Western blots analysis of H2AX, γH2AX, CHK2, p-CHK2 levels in Caco-2 cells from sgNC and sgEpCAM groups. n=6 per group. Lower panels: quantification data. **P<0.01 compared with sgNC group. sgNC, non-specific small guide RNA control; sgEpCAM, EpCAM knockout via CRISPR/Cas9 technology.


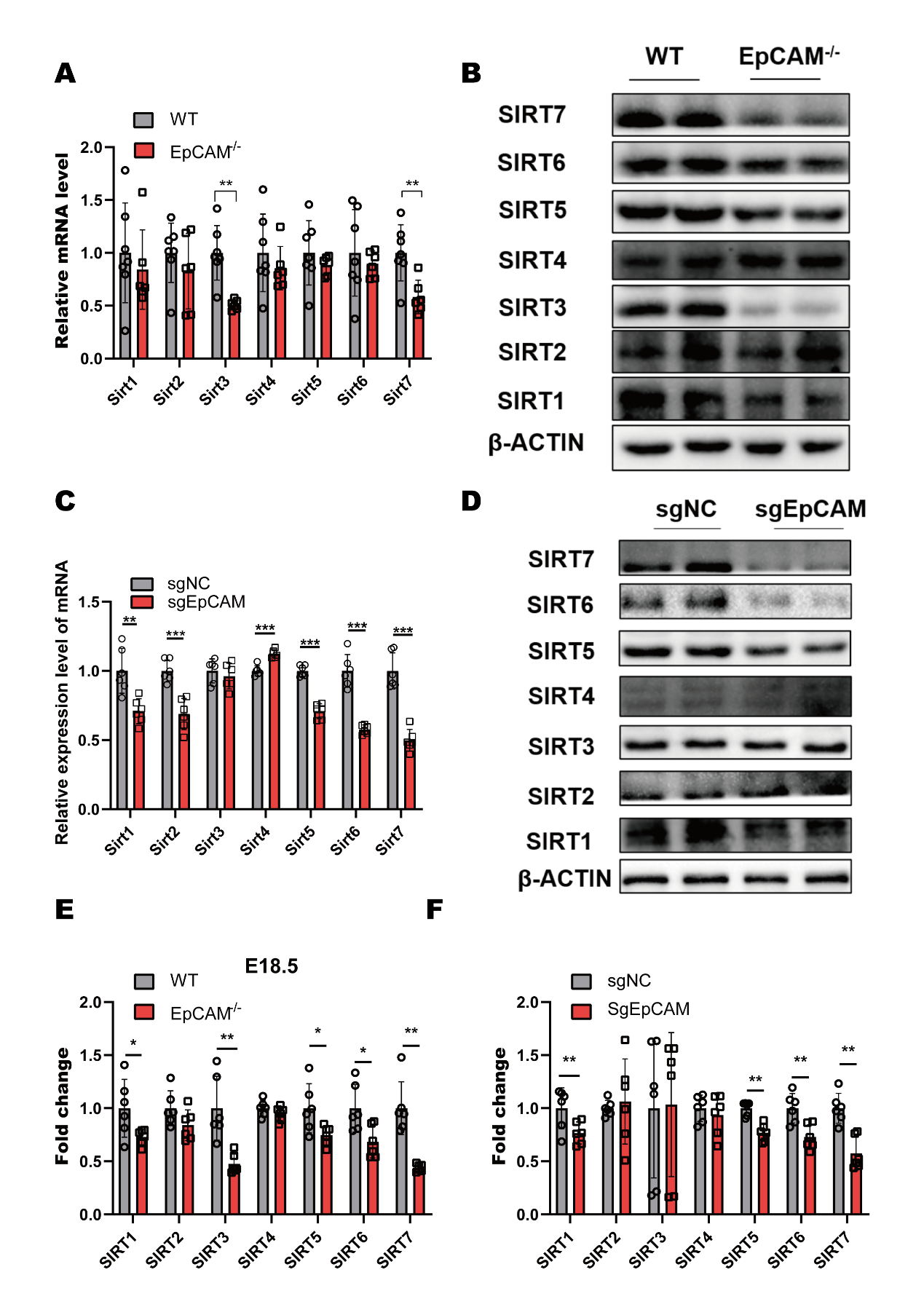


**Figure S4. EpCAM Deficiency Affected the Expression of Members of Sirtuin Family in the Intestines of E18.5 Embryonic Mice and Caco-2 Cells**

**A**. Relative mRNA expression levels of Sirt1-7 in the small intestines of WT and EpCAM^-/-^ mice at E18.5 stage (WT, n=7; EpCAM^-/-^, n=6.). **B**. Western blot results show the protein levels of SIRT1-7 in the small intestines from WT and EpCAM^-/-^ mice at E18.5 stage. **P<0.01 compared with WT group. **C**. The mRNA expression levels of Sirt1-7 in Caco-2 cells from sgNC and sgEpCAM groups (n=6 per group). ***P<0.001, **P<0.01 compared with sgNC group. **D**. Representative western blot images of SIRT1-7 in Caco-2 cells from sgNC and sgEpCAM groups. **E**. Quantification data of the western blot results of B. *P<0.05, **P<0.01 compared with WT group. **F**. Quantification data of the western blot results of D. **P<0.01 compared with sgNC group. sgNC, non-specific small guide RNA control; sgEpCAM, EpCAM knockout via CRISPR/Cas9 technology.


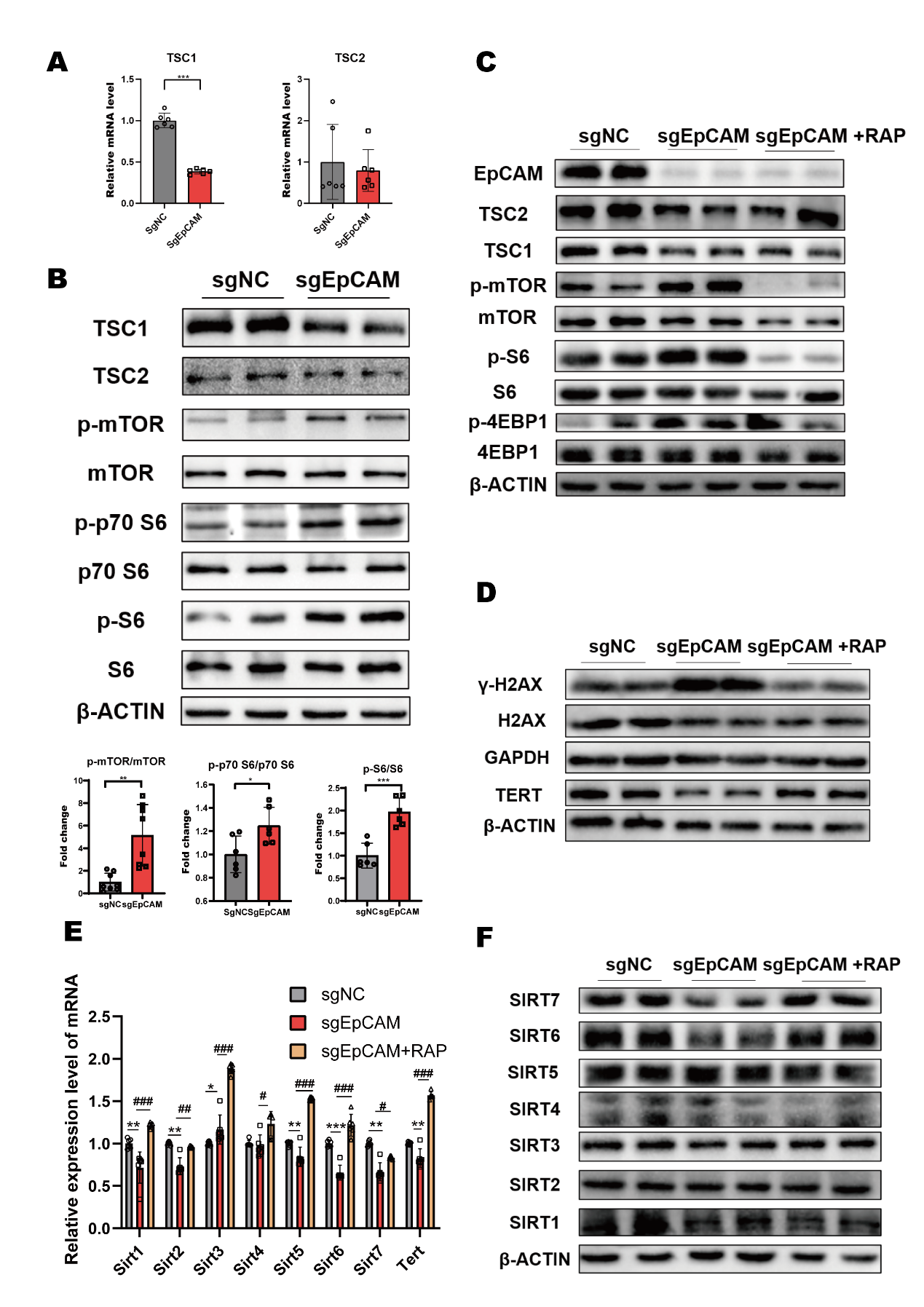


**Figure S5. Hyperactivation of mTORC1 Induced the Premature Aging of EpCAM**^-/-^ **Caco-2 Cells**

**A**. The mRNA expression levels of TSC1 and TSC2 in Caco-2 cells from sgNC and sgEpCAM groups (n=6 per group). ***P<0.001 compared with sgNC group. **B**. Western blots of TSC1, TSC2, p-mTOR, mTOR, p-p70S6, p70S6, p-S6 and S6 in Caco-2 cells from sgNC and sgEpCAM groups. Lower panels: quantification data (n=6 per group for 3 times independent experiments). ***P<0.001, **P<0.01 compared with sgNC group. **C**. Representative western blot images of EpCAM, TSC2, TSC1, p-mTOR, mTOR, p-S6, S6, p-4EBP1 and 4EBP1 in Caco-2 cells from sgNC, sgEpCAM and sgEpCAM +RAP groups (n=6 per group for 3 times independent experiments). **D**. Representative western blot images of γH2AX, H2AX and TERT in Caco-2 cells from sgNC, sgEpCAM and sgEpCAM +RAP groups (n=6 per group for 3 times independent experiments). **E**. The mRNA expression levels of Sirt1-7 and Tert in Caco-2 cells from sgNC, sgEpCAM and sgEpCAM +RAP groups (n=6 per group). **F**. Representative western blot images of SIRT1-7 in Caco-2 cells from sgNC, sgEpCAM and sgEpCAM +RAP groups (n=6 per group for 3 times independent experiments). ***P<0.001, **P<0.01, *P<0.05 compared with sgNC group; ^###^P<0.001, ^##^P<0.01, ^#^P<0.05 compared with sgEpCAM group. sgNC, non-specific small guide RNA control; sgEpCAM, EpCAM knockout via CRISPR/Cas9 technology; RPA, rapamycin.


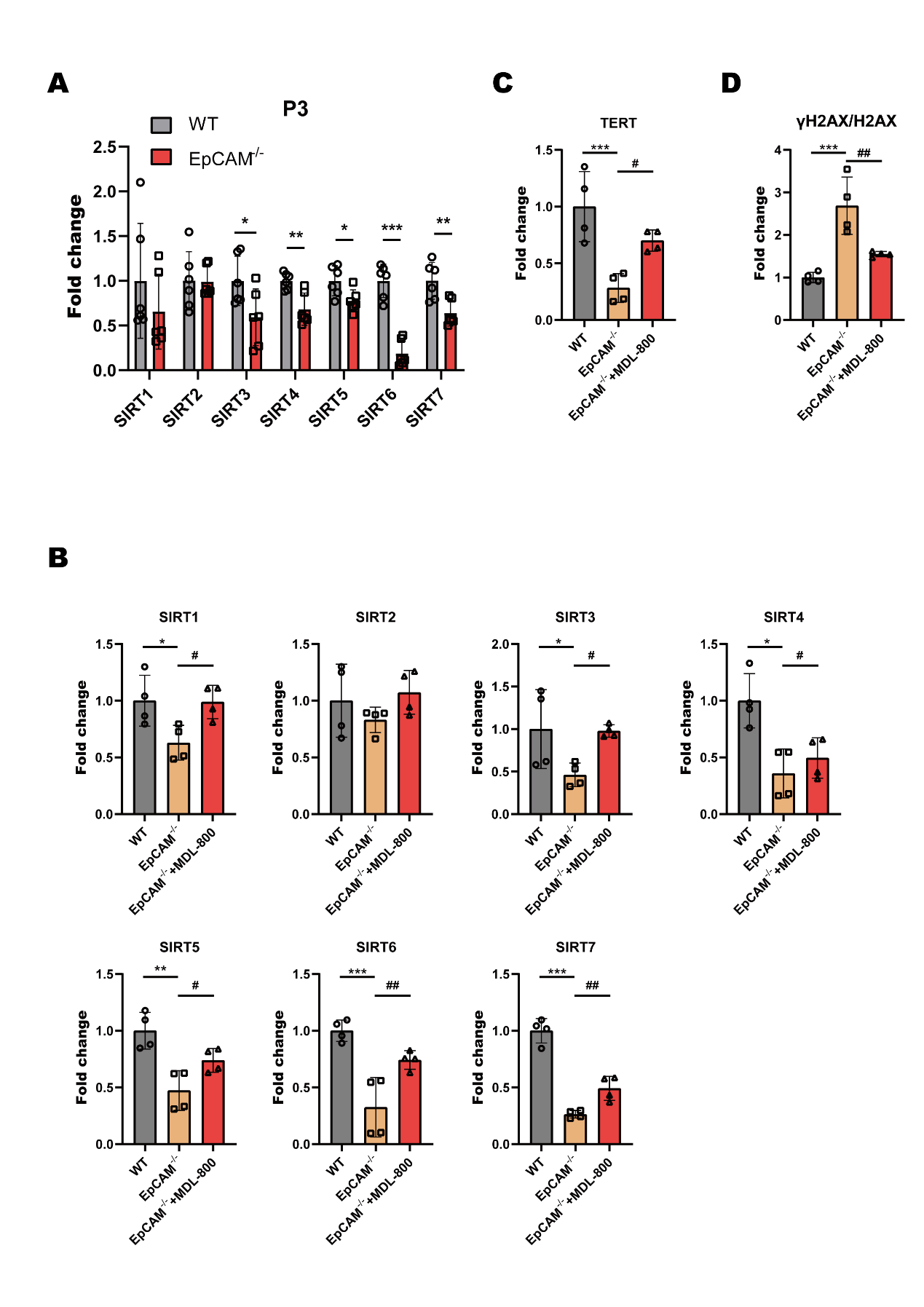


**Figure S6. Quantitative Analysis of the Western Blot Results in Figure 3**

**A-D**. Quantification data of the western blot results of Fig. 3B, D, F and G, respectively. A: 6 mice in each group for 3 times independent experiments; B-D: 4 mice in each group for 2 times independent experiments. *P<0.05, **P<0.01, ***P<0.001 compared with WT group; ^##^P<0.01, ^#^P<0.05 compared with EpCAM^-/-^ group.


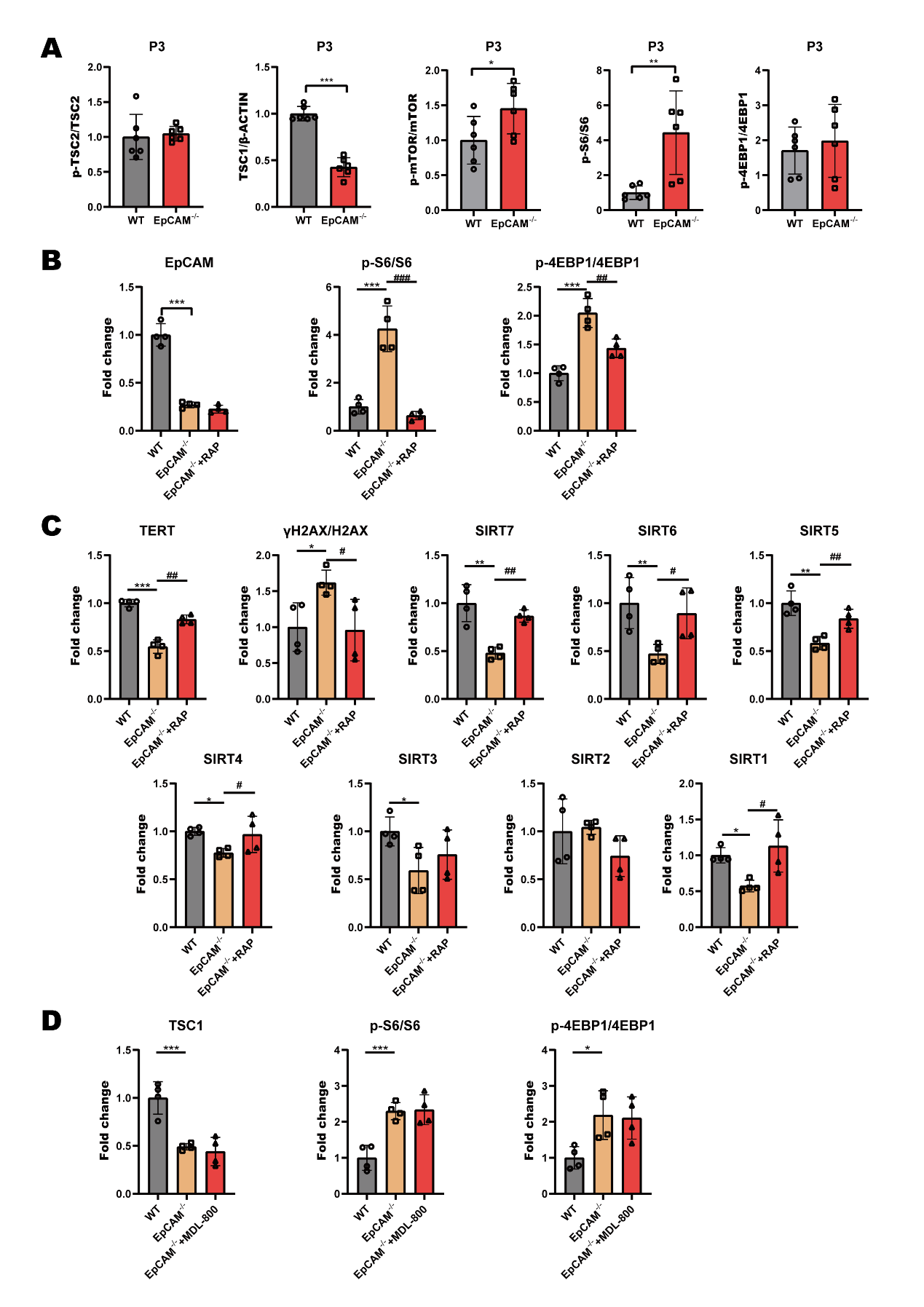


**Figure S7. Quantitative Analysis of the Western Blot Results in Figure 4**

**A-D**. Quantification data of the western blot results of Fig. 4B, E, G and H, respectively. A: 6 mice in each group for 3 times independent experiments; B-D: 4 mice in each group for 2 times independent experiments. *P<0.05, **P<0.01, ***P<0.001 compared with WT group; ^###^P<0.001, ^##^P<0.01, ^#^P<0.05 compared with EpCAM^-/-^ group.


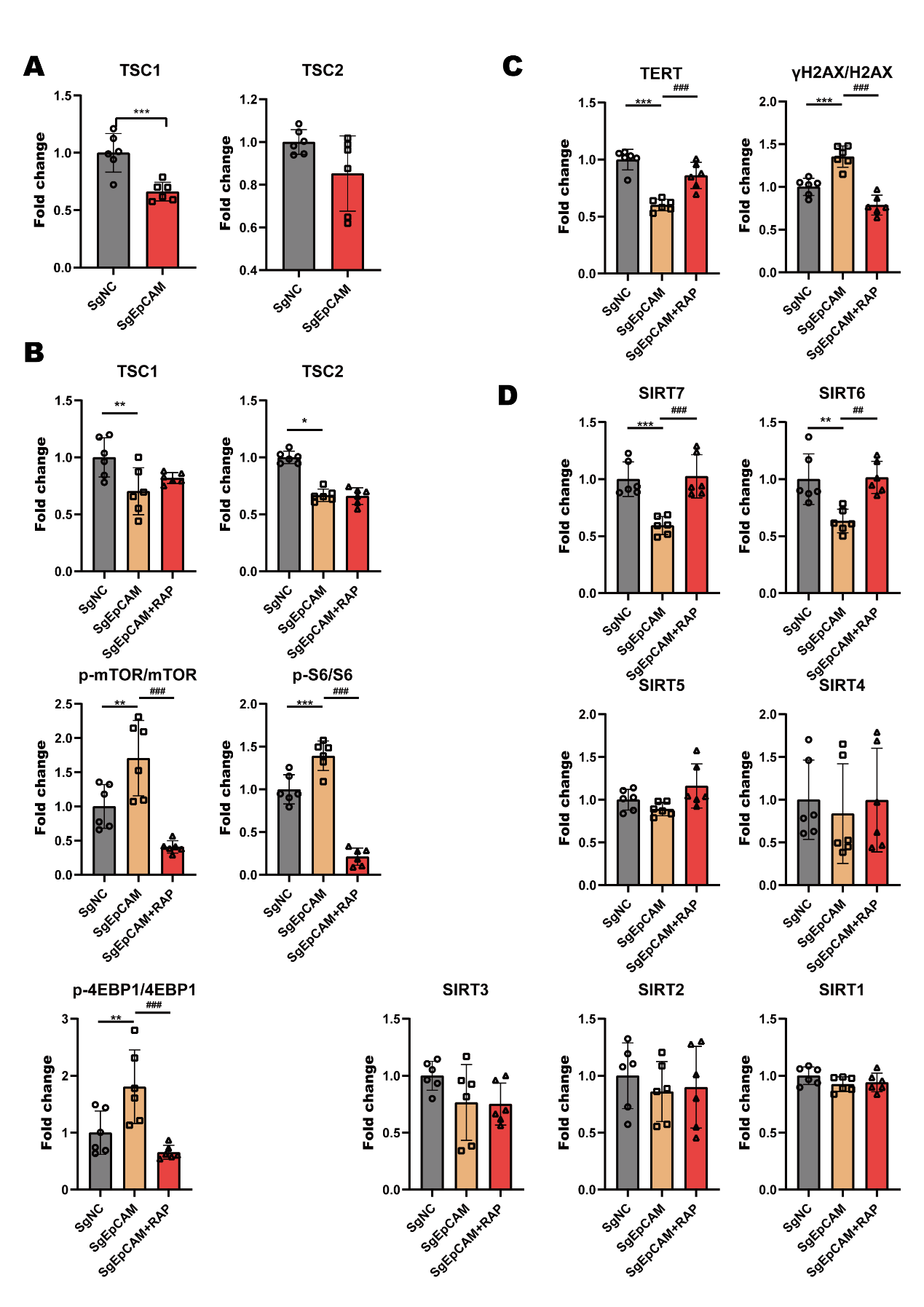


**Figure S8. Quantitative Analysis of the Western Blot Results in Figure S5**

**A-D**. Quantification data of the western blot results of Fig. S5B, C, D and F, respectively (n=6 per group for 3 times independent experiments). *P<0.05, **P<0.01, ***P<0.001 compared with sgNC group; ^###^P<0.001, ^##^P<0.01 compared with sgEpCAM group.

**Table S1. Sequences of primers used for qPCR**


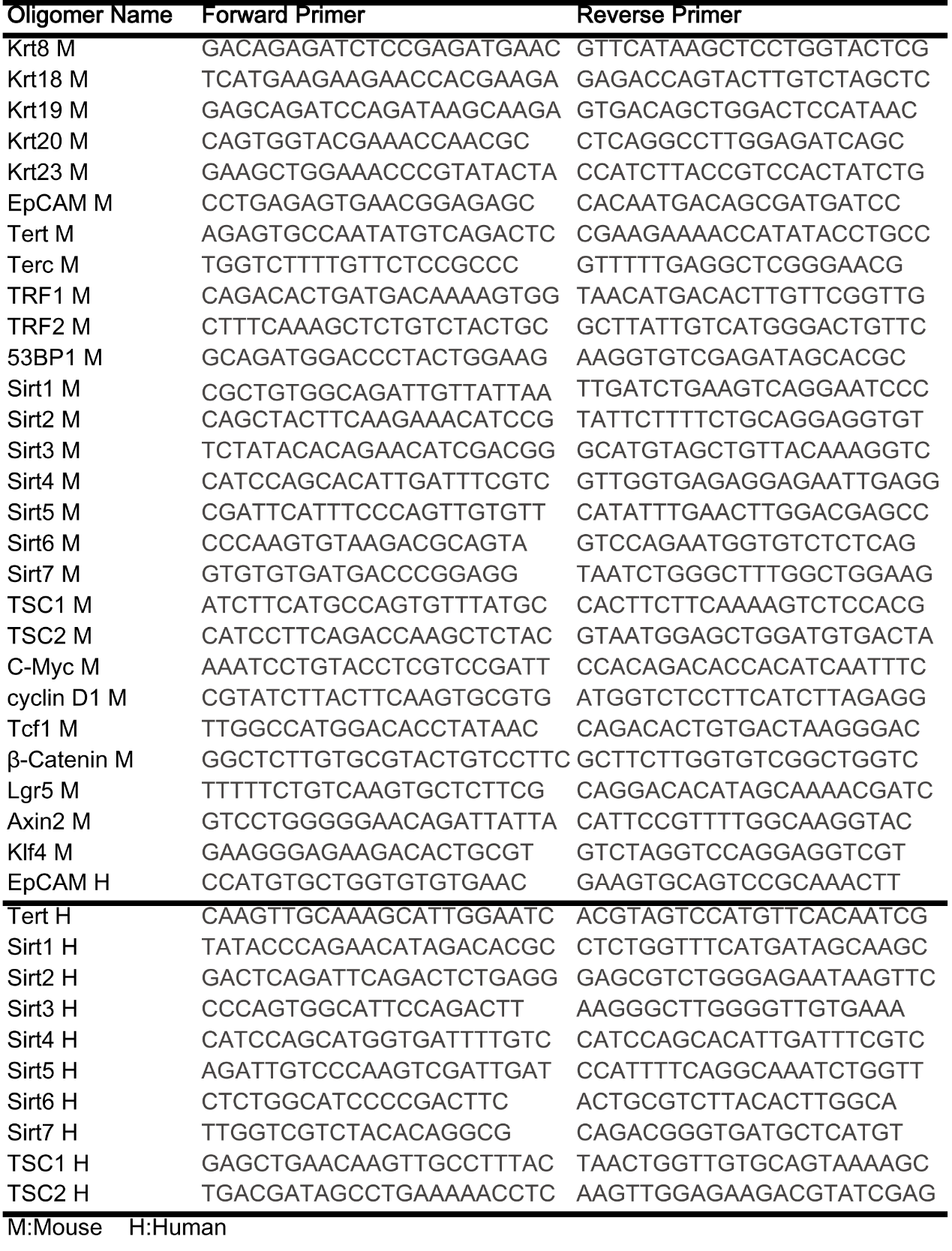


**Table S2. The primary and secondary antibodies used for western blot**


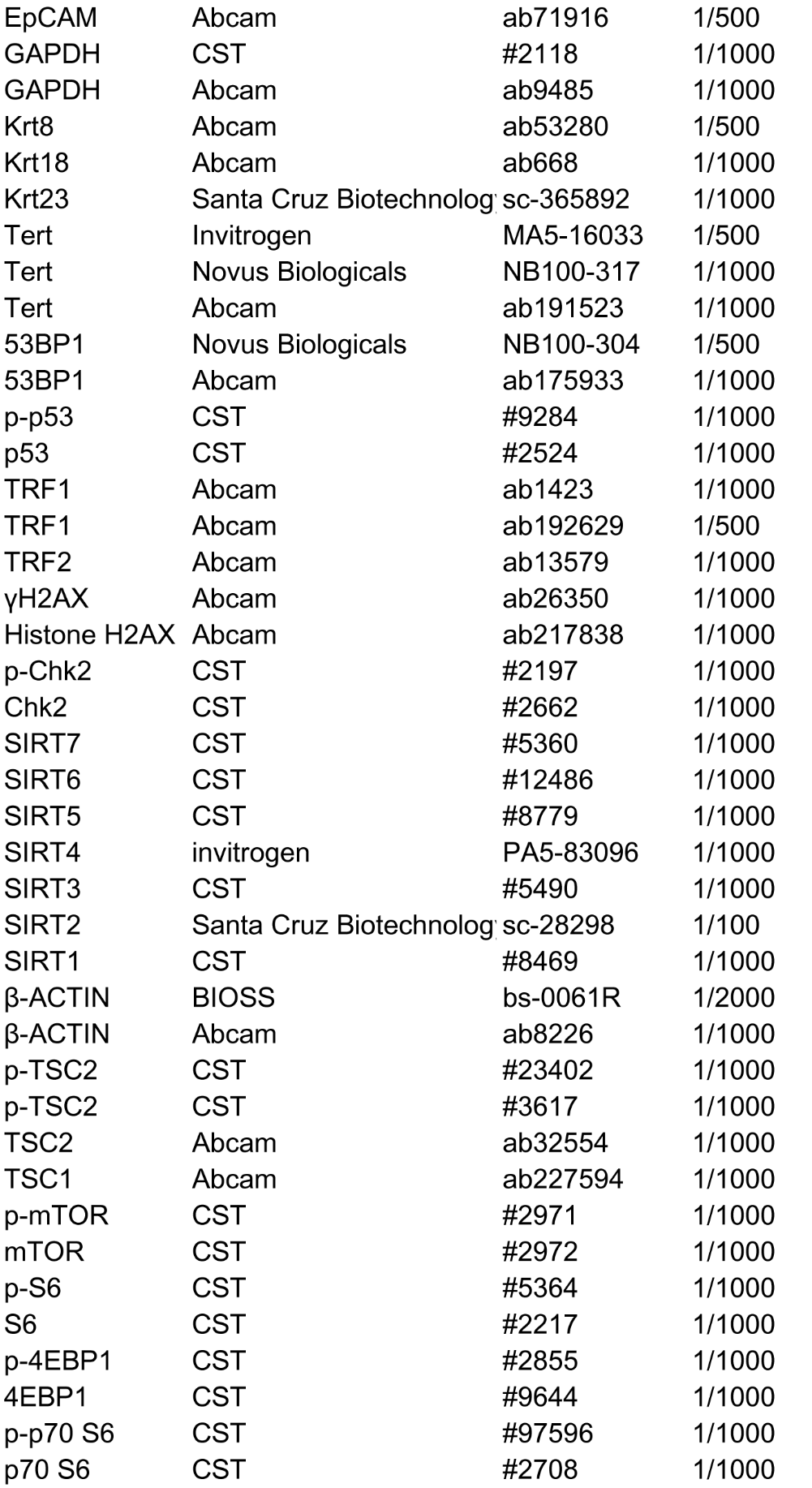

Supplement: Supplementary file 1 — Figure S1 EpCAM was successfully knockout in the intestinal epithelial cells both in vivo and in vitro Figure S2 EpCAM deficiency affected the compositions of telomerase and telomeres in the intestinal epithelial cells both in vivo and in vitro Figure S3 EpCAM deficiency caused accumulation of unrepaired DNA double‐strand breaks in caco‐2 cells Figure S4 EpCAM deficiency affected the expression of members of sirtuin family in the intestines of E18.5 embryonic mice and caco‐2 cells Figure S5. Hyperactivation of mTORC1 induced the premature aging of EpCAM‐/‐ Caco‐2 cells Figure S6 Quantitative analysis of the western blot results in Figure 3 Figure S7 Quantitative analysis of the western blot results in Figure 4 Figure S8 Quantitative analysis of the western blot results in Figure S5 Table S1 Sequences of primers used for qPCR Table S2 The primary and secondary antibodies used for western blot [file CTM2-12-e903-s001.docx]
